# The Spread and Transmission of Sweet Potato Virus Disease (SPVD) and Its Effect on the Gene Expression Profile in Sweet Potato

**DOI:** 10.3390/plants9040492

**Published:** 2020-04-10

**Authors:** Kai Zhang, Huixiang Lu, Chuanfang Wan, Daobin Tang, Yong Zhao, Kai Luo, Shixi Li, Jichun Wang

**Affiliations:** 1 College of Agronomy and Biotechnology, Southwest University, Beibei, Chongqing 400715, China; luhuixiangswu@163.com (H.L.); chuanfang2015@126.com (C.W.); tdbin741023@163.com (D.T.); zhy2419@126.com (Y.Z.); luokai1112@126.com (K.L.); swulsx@163.com (S.L.); 2 Key Laboratory of Biology and Genetic Breeding for Tuber and Root Crops in Chongqing, Beibei, Chongqing 400715, China; 3 State Cultivation Base of Crop Stress Biology for Southern Mountainous Land of Southwest University, Beibei, Chongqing 400715, China; 4 The Agricultural Science Research Institute of Liupanshui, Guizhou 553001, China

**Keywords:** virus, symptom, inoculation, metabolic pathway, control strategy

## Abstract

Sweet potato virus disease (SPVD) is the most devastating viral disease in sweet potato (*Ipomoea batatas* (L.) Lam.), causing substantial yield losses worldwide. We conducted a systemic investigation on the spread, transmission, and pathogenesis of SPVD. Field experiments conducted over two years on ten sweet potato varieties showed that SPVD symptoms first occurred in newly developed top leaves, and spread from adjacent to distant plants in the field. The SPVD incidence was mainly (but not only) determined by the resistance of the varieties planted, and each variety exhibited a characteristic subset of SPVD symptoms. SPVD was not robustly transmitted through friction inoculation, but friction of the main stem might contribute to a higher SPVD incidence rate compared to friction of the leaf and branch tissues. Furthermore, our results suggested that SPVD might be latent in the storage root. Therefore, using virus-free storage roots and cuttings, purposeful monitoring for SPVD according to variety-specific symptoms, and swiftly removing infected plants (especially during the later growth stages) would help control and prevent SPVD during sweet potato production. Comparative transcriptome analysis revealed that numerous genes involved in photosynthesis, starch and sucrose metabolism, flavonoid biosynthesis, and carotenoid biosynthesis were downregulated following SPVD, whereas those involved in monolignol biosynthesis, zeatin biosynthesis, trehalose metabolism, and linoleic acid metabolism were upregulated. Notably, critical genes involved in pathogenesis and plant defense were significantly induced or suppressed following SPVD. These data provide insights into the molecular changes of sweet potato in response to SPVD and elucidate potential SPVD pathogenesis and defense mechanisms in sweet potato. Our study provides important information that can be used to tailor sustainable SPVD control strategies and guide the molecular breeding of SPVD-resistant sweet potato varieties.

## 1. Introduction

Sweet potato is an important and versatile crop that is widely grown throughout the world as an edible tuber, a green vegetable, a nutritional supplement, a livestock feed, an ornamental plant, an industrial raw material, and a potential bioethanol feedstock [1,2]. Sweet potato’s high yield potential and adaptability to a wide range of environmental conditions make it an important staple and food security crop in many regions of the world, especially in developing countries [3].

The most devastating disease in sweet potato globally is sweet potato virus disease (SPVD), which is caused by co-infection by aphid-borne *sweet potato feathery mottle virus* (SPFMV) and whitefly-borne *sweet potato chlorotic stunt virus* (SPCSV) [4]. Symptoms of SPVD include dwarfing, vein clearing, leaf chlorosis, distortion, puckering, stunting, discoloration, and deformity. SPVD can cause yield losses of 90% and poses a significant constraint on sweet potato production [5,6].

A series of control measures have been developed to prevent SPVD in sweet potato production. These measures mainly focus on breeding and releasing SPVD-resistant or -tolerant varieties, establishing isolated sites and nurseries for production of virus-free planting materials, using virus-free seed and seedlings and implementing vector control programs. An effective strategy for preventing further spread of the virus is the timely removal of plants with SPVD in the field [4]. Although these strategies have been used in major sweet potato production areas, SPVD remains a challenging disease to manage. Understanding the epidemiology of SPVD is critical for tailoring and implementing strategies for its management.

SPFMV is a member of the genus *Potyvirus* in the Potyviridae family, while SPCSV belongs to the genus *Crinivirus* in the Closteroviridae family [4]. Both viruses are single-stranded RNA (ssRNA) viruses [7,8,9]. Based on analysis of the complete genomic sequences of virus isolates, three representative strains of SPFMV—the russet crack (RC), ordinary (O), and East Africa (EA) groups—have been recognized. Two distinct strain groups have been recognized for SPCSV [9,10]—the East African (EA) and West African (WA) groups [5,7].

Individually, SPFMV causes mild or no symptoms, while SPCSV can lead to mild to moderate symptoms of overall chlorosis, slight stunting and purpling of older leaves, and mild chlorotic mottle in the middle leaves, with yield losses of up to 43% [7,11]. However, dual infection by the two viruses causes a dramatic increase in the titer of SPFMV, increasing the severity of disease symptoms and yield loss [6,12]. The titer of SPFMV can increase by up to 600-fold in co-infected plants compared with those infected with SPFMV alone, whereas the titer of SPCSV is not significantly altered in the co-infected plants [7,11]. Previous studies have shown that resistance to SPFMV can be broken when plants are co-infected with SPCSV. Transformation of the sweet potato variety “Huachano”, which is extremely resistant to SPFMV, with the double-stranded RNA (dsRNA)-specific class 1 RNA endoribonuclease III (RNase3) of SPCSV broke down resistance to SPFMV, leading to high titers of SPFMV and severe SPVD symptoms [8].

To explore the mechanisms underlying the synergism between SPFMV and SPCSV and the effect on plant defenses, Kokkinos et al. [12] used cDNA microarrays to identify genes that are differentially expressed in virus-inoculated versus non-inoculated plants. Compared with healthy plants, 3, 14, and 216 genes were differentially expressed in the leaves of plants graft-inoculated with SPFMV-RC alone, SPCSV alone, and both viruses combined, respectively. Several plant resistance (R) genes, pathogenesis-related (PR) genes, and heat shock protein 70 (HSP70) genes were identified as being important for SPVD defense in sweet potato.

Efforts have been made to select varieties resistant to SPVD. Genetic relationships of sweet potato genotypes from different origins were evaluated using simple sequence repeat (SSR) markers [13]. The genetic diversity of sweet potato accessions was evaluated on different ploidy levels, using SSR markers and two high-resolution capillary platforms [14]. These results provide baseline data for resistant genotype selection. Seven SSR markers significantly associated with SPVD resistance were identified in a biparental sweet potato mapping population [15] and genotypes resistant to SPVD have been selected in recent years [16,17,18]. However, until recently, limited information has been available on the spread and transmission mechanisms of SPVD, or on the interaction between sweet potato and SPVD viruses in the field.

In this study, we investigated the spread of SPVD under natural conditions over two years of field experiments and used friction and graft inoculation to examine the mechanism of SPVD transmission. We also carried out comparative transcriptome analysis to elucidate the interaction between SPVD and the infected plants and to identify potential resistance mechanisms. Our results provide insights for monitoring and preventing SPVD in sweet potato production, breeding of resistant varieties, and for further investigation of virus defense mechanisms in sweet potato.

## 2. Results

### 2.1. Characteristics of SPVD Spread in the Field

**Figure 1:**
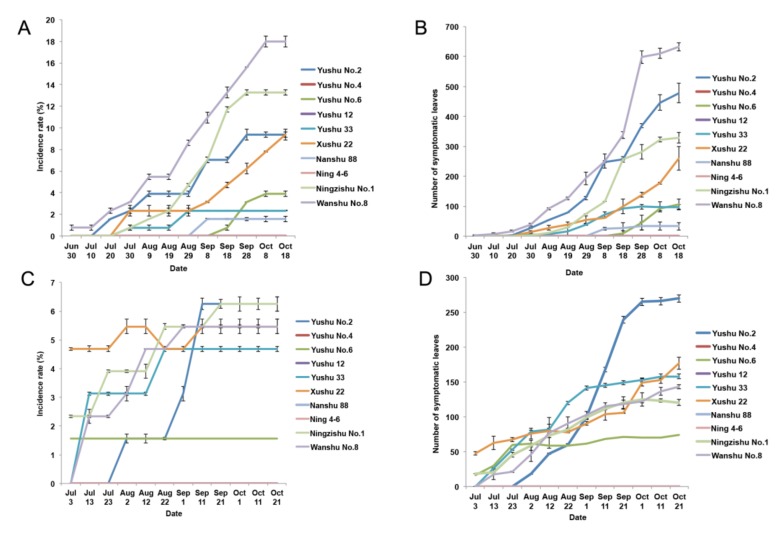
Sweet potato virus disease (SPVD) incidence in 10 sweet potato varieties in the field experiment. (**A**) SPVD incidence rate calculated during the whole experiment period in 2014. (**B**) The number of symptomatic leaves recorded in 2014. (**C**) SPVD incidence rate calculated during the whole experiment period in 2015. (**D**) The number of symptomatic leaves recorded in 2015. Error bars indicate the standard deviation from two independent replicates.

To assess the spread mechanism of SPVD under natural conditions, we conducted field experiments in 2014 and 2015. No large-scale dispersal of SPVD was detected in these experiments in either year. In 2014, there was no transmission of SPVD between plants of Yushu No.4, Yushu 12, and Ning 4-6 sweet potato varieties in the two replicated experimental plots. Natural SPVD transmission did occur between plants of the other varieties, and the incidence rate (the percentage of SPFMV and SPCSV-infected plants) increased over the course of the growth season. The incidence rate ranged from 0% to 15% in the 20 experimental plots (Figure 1A).

Because the low disease incidence rate might not fully reflect the virus transmission, we also counted the numbers of SPFMV and SPCSV-infected leaves in each experimental plot (Figure 1B). The number of infected leaves ranged from 0 to 645 in the 20 experimental plots. Plants of Wanshu No.8 variety had the highest incidence rate among the tested varieties at all 12 time points. From July 30 to October 18, the average incidence rate for Wanshu No.8 increased from 0.78% to 17.97% and the number of infected leaves increased from 3 to 632. Ningzishu No.1, Yushu No.2, and Xushu 22 plants also showed a large increase in the number of infected plants and leaves over the course of the experiment. Low incidence rates (0 to 3.91%) and infected leaf numbers (0 to 106) were detected for Yushu No.6, Yushu 33, and Nanshu 88 plants.

The field experiment was replicated in 2015, and the SPVD incidence rates were much lower than in 2014 (Figure 1C,D). There were no newly infected plants in either replicate plot for Yushu No.4, Yushu 12, Ning 4-6, and Nanshu 88 varieties. For Yushu No.6, newly infected plants appeared in only one of the two replicate plots. The number of infected plants and leaves increased during the first seven time points but did not increase thereafter. Similar to the results obtained in 2014, Wanshu No.8, Ningzishu No.1, Yushu No.2, and Xushu 22 plants had high or moderate incidence rates and a rapid increase in the number of infected leaves. However, Yushu 33 plants, which had a low incidence rate in 2014, showed a rapid increase in the number of infected plants and leaves in 2015. Another difference from 2014 was that Yushu No.2, not Wanshu No.8, had the highest level of infection among the tested varieties, with a 6.25% incidence rate and 270 infected leaves.

**Figure 2:**
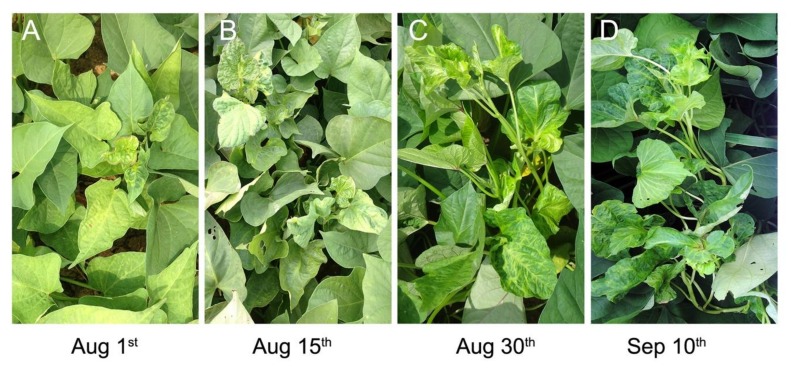
SPVD symptoms exhibited by Wanshu No.8 plants. SPVD symptoms first developed in the top leaves (**A**) and then leaves throughout the plant showed distortions or chlorotic symptoms (**B**–**D**). The photos were taken on August 1st (**A**), August 15th (**B**), August 30th (**C**), and September 10th (**D**) during the field experiments performed in 2014, respectively.

Over the course of the field experiments, the symptoms of SPVD generally first appeared on plants near the primary infected plants in the center of the plots. The SPVD symptoms generally appeared on the young leaves at the top of the plant first (Figure 2), in agreement with a previous report that SPVD developed first in the newly emerging leaves [11,12]. Eventually, the symptoms spread throughout the plants, which became dwarfed and had distorted or chlorotic leaves (Figure 2). Finally, plants in the vicinity of these infected plants also became infected. The spread of SPVD was relatively rapid during the seedling growth stage but there was no large-scale dispersal of SPVD at later stages of plant growth, especially in 2015. Although the number of SPFMV and SPCSV-infected plants did not increase rapidly, the number of infected leaves did, indicating that transmission of virus within a plant is faster than that from plant to plant in the field.

**Figure 3:**
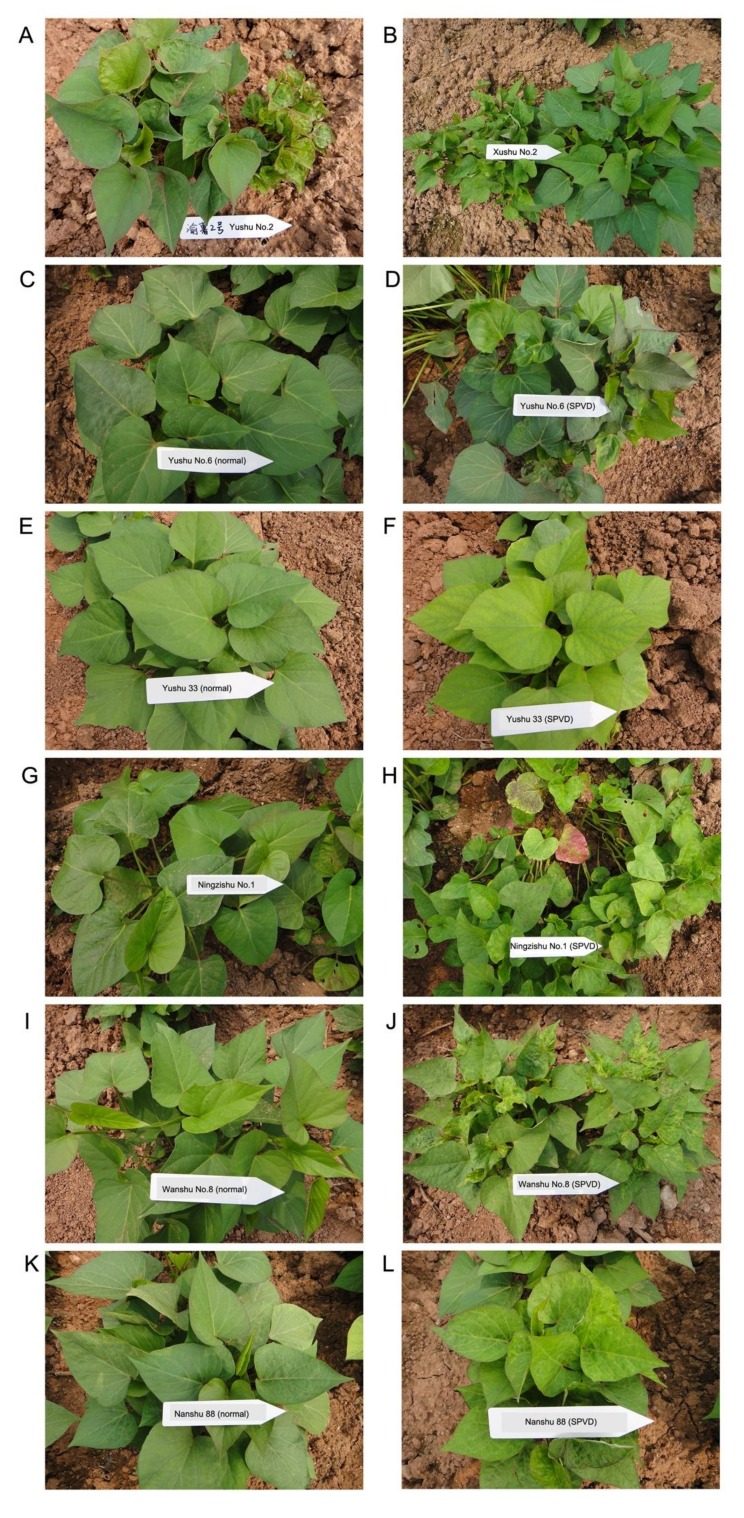
Characteristic SPVD symptoms in different sweet potato varieties: (**A**) uninfected Yushu No.2 plants (left) and Yushu No.2 plants co-infected by *sweet potato feathery mottle virus* (SPFMV) and *sweet potato chlorotic stunt virus* (SPCSV) (right); (**B**) uninfected (right) and infected (left) Xushu 22 plants; uninfected (**C**) and infected (**D**) Yushu No.6 plants; uninfected (**E**) and infected (**F**) Yushu 33 plants; uninfected (**G**) and infected (**H**) Ningzishu No.1 plants; uninfected (**I**) and infected (**J**) Wanshu No.8 plants; uninfected (**K**) and infected (**L**) Nanshu 88 plants. All the photos were taken at seeding stage to exhibit the symptoms of the whole plants.

A wide range of typical SPVD symptoms could be observed in the field experiments, and the symptoms varied among the 10 varieties and showed slight differences from plant to plant within each variety. Variety-specific symptoms at the whole-plant level are shown for SPFMV and SPCSV-infected seedlings in Figure 3. Infected Yushu No.2 showed dwarfing, leaf chlorosis, leaf distortion, and bright veins (Figure 3A). In plants of Xushu 22, SPFMV and SPCSV infection caused dwarfing, yellowing, and small leaves (Figure 3B). Leaf distortion without chlorosis was the most obvious symptom of infected plants of Yushu No.6 (Figure 3D), but in Yushu 33, infected leaves showed chlorosis and yellowing but not distortion (Figure 3F). Ningzishu No.1 is a purple-fleshed variety, but its leaves are green (Figure 3G). After infection, the Ningzishu No.1 leaves exhibited serious distortion, yellow and purple mottling, or purple margins (Figure 3H). Infected Wanshu No.8 plants exhibited dwarfing, leaf crinkle, distortion, chlorosis, and yellowing (Figure 3J), while those of Nanshu 88 plants showed leaf chlorosis and bright veins (Figure 3L).

In summary, symptoms first appear on the top young leaves and then spread throughout the plant, from adjacent to distant plants. The infection spreads rapidly in young seedlings but slows during later growth stages, and the particular symptoms displayed depend on the sweet potato varieties.

### 2.2. SPVD Transmission through Artificial Friction Inoculation

**Figure 4:**
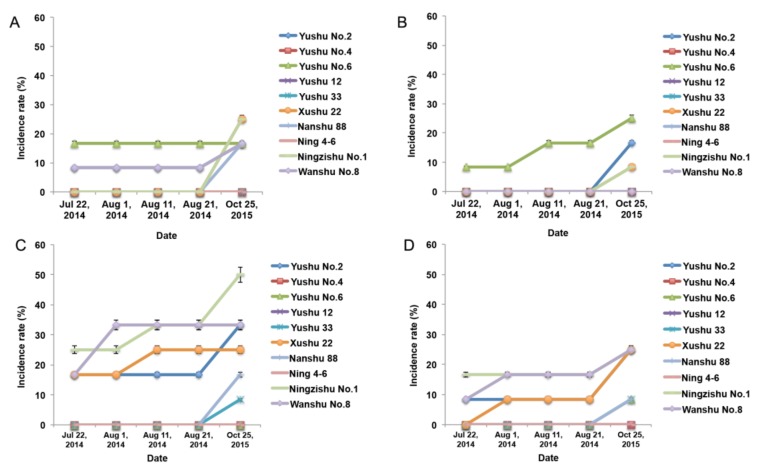
SPVD incidence rates through friction inoculation. SPVD incidence rates of 10 sweet potato varieties through friction inoculation of top leaves (**A**), branch leaves (**B**), stems (**C**), and branches (**D**). Error bars represent standard errors. The friction inoculation was performed twice in July, 2014. The number of symptomatic plants was recorded at 9-day intervals from the date on which the first symptomatic plant was detected. The storage roots were collected and planted in 2015 to further survey the latent virus.

To elucidate the transmission of SPVD and to determine which organs of sweet potato are most susceptible to SPFMV and SPCSV infection, artificial friction was used to inoculate leaves, stems, branches, and branch leaves of the 10 sweet potato varieties. The highest incidence rate in these experiments was 50%, indicating that SPVD is not transmitted well by friction inoculation (Figure 4). However, friction inoculation of the top and branch leaves resulted in 8.33% to 25% SPFMV and SPCSV infection rates in Yushu No.6 plants (Figure 4A,B). Compared to leaf, branch, and branch leaf inoculation, friction inoculation of stems generated more infected plants (Figure 4C), indicating that the main stem is more susceptible to SPFMV and SPCSV infection compared to other organs.

To further test the effect of friction inoculation on virus transmission in these plants, we harvested the storage roots of plants that had no obvious SPVD symptoms and were diagnosed as uninfected plants. Tips generated from the storage roots were then planted in 2015 in the same way. The number of SPFMV and SPCSV-infected plants was higher in 2015 than it had been in 2014, regardless of which organ had been friction-inoculated originally (Figure 4), indicating that the virus was actually transmitted through friction inoculation and was latent in storage roots.

### 2.3. Grafting was Effective for SPVD Transmission

Since friction inoculation was not very successful, we used graft inoculation to produce systemic SPFMV and SPCSV infections in 7 of the 10 sweet potato varieties in 2016. As shown in Table 1, the disease indices of the grafted plants ranged from 2.86 to 35.00 at 15 days after grafting (DAG) and from 5.71 to 75.00 at 30 DAG. Ning 4-6 had the highest disease index at 15 DAG and Ningzishu No.1 had the highest at 30 DAG. Yushu No.4, Yushu No.6, and Yushu 12 had low disease indices in the grafting test, and nitrocellulose membrane enzyme-linked immunosorbent assay (NCM-ELSA) and qRT-PCR showed that these plants were uninfected by SPFMV or SPCSV. The disease indices rapidly increased from 15 DAG to 30 DAG for Xushu 22, Ning 4-6, and Ningzishu No.1 plants. Our results show that systemic infection through grafting was a more effective method than friction for SPVD transmission in sweet potato.

### 2.4. Comparison of the Transcriptomes of Non-Infected and SPFMV and SPCSV-Infected Sweet Potato

To decipher the pathogenesis of SPVD and to investigate the responses of sweet potato plants during natural SPVD transmission, we compared the transcriptomes of SPFMV and SPCSV-infected (S14) and non-infected (H14) Wanshu No.8 plants collected from our field experiments. We obtained 59,596,538 raw reads for H14 and 73,348,562 for S14, for a total of 5.96×.9^10^ and 7.33×10^10^ bases, respectively. After trimming the adaptor sequences and removing sequences with unknown or low quality bases, approximately 2.98×10^8^ and 3.67×10^8^ sequences (92.34% and 94.14% of the Hiseq2000 sequence reads, without keys, tags, or poor quality bases) were assembled using Trinity into 47,154 unigenes for H14 and 46,823 unigenes for S14. The sequencing data were deposited in the BIG Data Center under BioProject accession code PRJCA002291.

**Figure 5:**
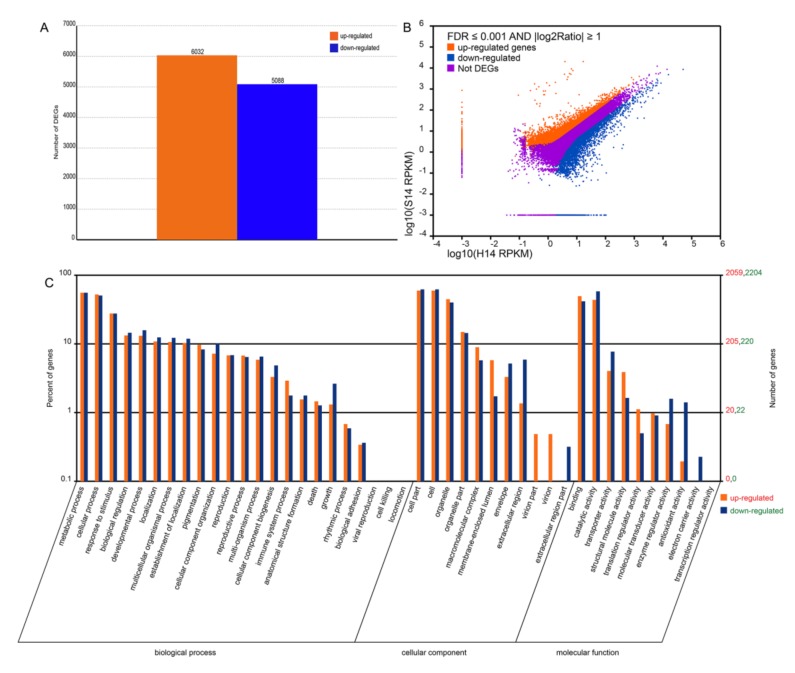
Comparison of the transcriptomes of non-infected and SPFMV and SPCSV-infected sweet potato plants H14 and S14. (**A**) The number of differentially expressed genes (DEGs) between non-infected (H14) and infected sweet potato plants (S14). The height of the bar shows the number of genes that are differentially expressed between H14 and S14. Upregulated expression is indicated in orange and downregulated expression in blue. (**B**) Gene expression levels in H14 and S14. Upregulated genes are shown as orange bars, downregulated genes as blue bars, and non-DEGs as purple bars. (**C**) Gene ontology (GO) categories of the DEGs. The heights of the bars show the number of DEGs in the specified GO categories. Upregulated categories are presented in orange and downregulated categories in blue.

A total of 11,120 genes were identified as being differentially expressed between H14 and S14. Relative to H14, 6032 unigenes were upregulated and 5088 were downregulated in S14 (Figure 5A,B), indicating that SPFMV and SPCSV infection greatly affected the gene expression profile in sweet potato.

### 2.5. Gene Ontology and Metabolic Pathway Analysis of Differentially Expressed Genes

The functional classes of the differentially expressed genes (DEGs) were subjected to gene ontology (GO) enrichment analysis. Out of the 11,120 DEGs, 9153 (82.31%) could be annotated with GO terms. A large number of DEGs fell into the following categories: metabolic process, cellular process, and response to stimulus within the biological process (BP) class; cell part and cell and organelle within the cellular component (CC) class; and binding and catalytic activity within the molecular function (MF) class (Figure 5C).

As expected, all DEGs in the virion part and virion categories were upregulated in S14 relative to H14. All DEGs in the extracellular region part and electron carrier activity categories were downregulated. In the categories of immune system process, death, pigmentation, rhythmic processes, macromolecular complex, membrane-enclosed lumen, binding, structural molecular activity, and translation regulator activity, there were more upregulated genes than downregulated genes. However, the opposite was true for the cellular component organization, developmental process, growth, envelope, extracellular region, catalytic activity, transporter activity, enzyme regulator activity, and antioxidant activity categories, which had more downregulated than upregulated genes (Figure 5C).

**Figure 6:**
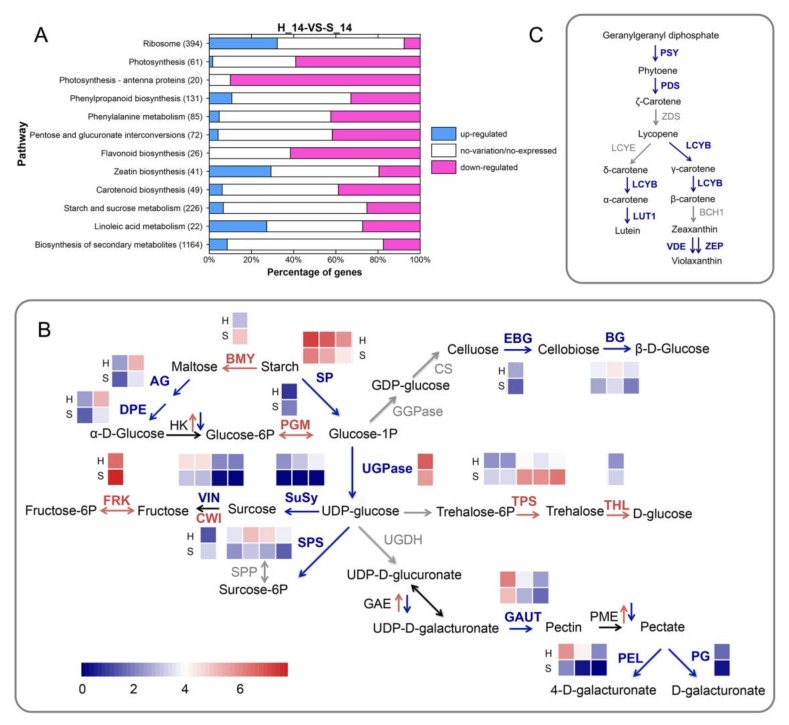
Differentially expressed genes (DEGs) involved in biological pathways. (**A**) The top 12 metabolic pathways with significant enrichment of DEGs. The numbers in parentheses represent the numbers of DEGs with pathway annotations. (**B**) Illustration of the enzymes encoded by DEGs involved in carbohydrate metabolism. Note: AG, alpha-glucosidase (EC 3.2.1.20); BG, β-glucosidase (EC 3.2.1.21); BMY, β-amylase (EC 3.2.1.2); CWI, cell wall invertase; CS, cellulose synthase (guanosine diphosphate (GDP)-forming) (EC 2.4.1.29); DPE, 4-α-glucanotransferase (EC 2.4.1.25); EBG, endo-1, 4-beta-glucanase (EC 3.2.1.4); FRK, fructokinase (EC 2.7.1.4); GAE, UDP-glucuronate 4-epimerase (EC 5.1.3.6); GAUT, galacturonosyltransferase (EC 2.4.1.43); GGPase, GDP glucose pyrophosphorylase (EC 2.7.7.34); HK, hexokinase (EC 2.7.1.1); PEL, pectate lyase (EC 4.2.2.2); PG, polygalacturonase (EC 3.2.1.15); PGI, glucose-6-phosphate isomerase (EC 5.3.1.9); PGM, phosphoglucomutase (EC 5.4.2.2); SP, starch phosphorylase (EC 2.4.1.1); SPP, sucrose phosphate phosphatase (EC 3.1.3.24); SuSy, sucrose synthase (EC 2.4.1.13); PME, pectin methyl esterase (EC 3.1.1.11); THL, trehalase (EC 3.2.1.28); TPS, α, α-trehalose-phosphate synthase (uridine diphosphate (UDP)-forming) (EC 3.1.3.12); UGDH, UDP-glucose 6-dehydrogenase (EC 1.1.1.22); UGPase, UDP-glucose pyrophosphorylase (EC 2.7.7.9); VIN, soluble acid invertase (EC 3.2.1.26). Red and blue fonts represent up- and downregulation of gene expression, respectively. Each box depicts an individual gene. Enzymes encoded by upregulated, downregulated, and non-differentially expressed genes are shown in red, blue, and gray, respectively. The scale bar represents reads per kilobase of exon model per million mapped reads (RPKM) values. (**C**) DEGs involved in carotenoid biosynthesis. Note: BCH1, carotenoid β-hydroxylase (EC 1.14.15.24); LCYB, lycopene β-cyclase (EC 5.5.1.19); LCYE, lycopene ε-cyclase (EC 5.5.1.18); LUT1, cytochrome P450-type monooxygenase (EC 1.14.14.158); PDS, phytoene desaturase (EC 1.3.5.5); PSY, phytoene synthase (EC 2.5.1.32); VDE, violaxanthin de-epoxidase (EC 1.10.99.3); ZDS, ζ-carotene desaturase (EC 1.14.99.30); ZEP, zeaxanthin epoxidase (EC 1.14.13.90). Enzymes encoded by non-differentially expressed and downregulated genes are shown in gray and blue, respectively.

To further elucidate the biological pathways affected by SPFMV and SPCSV infection, the DEGs were analyzed using the KEGG pathways database. A total of 2211 DEGs were assigned to 118 KEGG pathways. Of these, 10 pathways with *q* ≤ 0.01 and 2 pathways with *q* ≤ 0.05 were significantly enriched. There was a remarkable repression of genes related to photosynthesis (ko00195), flavonoid biosynthesis (ko00941), carotenoid biosynthesis (ko00906), and starch and sucrose metabolism (ko00500). By contrast, genes involved in ribosome (ko03010), zeatin biosynthesis (ko00908), and linoleic acid metabolism (ko00591) were induced in S14 (Figure 6A).

### 2.6. Genes Involved in Photosynthesis are Downregulated in SPFMV and SPCSV-Infected Plants when Compared with Non-Infected Plants

Transcription of genes related to photosynthesis was suppressed in SPFMV and SPCSV-infected plants relative to non-infected plants. Many photosystem II genes were downregulated, including most of those encoding chlorophyll a/b binding proteins and several of those encoding reaction center proteins. Several photosystem I reaction center subunit genes were also downregulated. Furthermore, genes encoding components of photosynthetic electron transport were significantly downregulated, including the cytochrome b6-f complex iron-sulfur subunit; ferredoxin-NADP^+^ reductase; and ATP synthase α (CF_1_), δ (CF_1_), and b’ (CF_0_) subunits.

### 2.7. SPVD Affects the Expression Levels of Genes Involved in Carbohydrate Metabolism

The DEGs encoding enzymes involved in starch and sucrose metabolism and in pentose and glucuronate interconversions are shown in Figure 6B. In comparison with non-infected plants, the infected plants showed increased expression of genes controlling trehalose metabolism but reduced expression of genes related to cellulose and pectin metabolism, including cell-wall-modifying enzyme, endo-1, and 4-beta-glucanase (EBG) encoding genes. Most of the genes involved in starch and sucrose metabolism were downregulated, particularly those related to glucose metabolism. With the exception of the β-amylase gene, which was upregulated, genes for starch degradation enzymes, including 4-α-glucanotransferase (DPE) and starch phosphorylase (SP), were downregulated.

Genes involved in sucrose cleavage, including those encoding sucrose synthase (SuSy) and UDP-glucose pyrophosphorylase (UGPase), also showed downregulated expression. Invertase genes also showed differential expression between non-infected and infected plants. A cell wall invertase unigene was upregulated in infected plants, but three vacuolar invertase genes, including the previously reported sweet potato vacuolar invertase genes *Ibβfruct2* and *Ibβfruct3* [19,20], were downregulated. Furthermore, genes for a plastid glucose transporter (GT) showed downregulated expression in the infected plants, and several other sugar transporter genes were DEGs. The SWEET sugar transporter gene *SWEET2*, *3*, *10*, and *11* homologs were downregulated, but the *SWEET1*, *12*, and *14* homologs were upregulated in the infected plants. These results indicated that SPVD greatly influenced the expression of genes encoding key enzymes and transporters involved in carbohydrate biosynthesis and metabolism.

### 2.8. Expression of Phenylpropanoid Metabolic Pathway Genes is Altered in the Infected Plants

Flavonoid metabolism and monolignol biosynthesis are two branches of the phenylpropanoid metabolic pathway. Comparative transcriptome analysis showed that after SPFMV and SPCSV infection, genes encoding key enzymes in flavonoid biosynthesis, including chalcone synthase (CHS), chalcone isomerase (CHI), anthocyanidin synthase (ANS), flavonol synthase (FLS), dihydroflavonol 4-reductase (DFR), flavanone 3-hydroxyrase (F3H), and flavonoid 3′-hydroxylase (F3′H), were downregulated. However, genes encoding ferulate-5-hydroxylase (F5H) and cinnamoyl-CoA reductase (CCR), which are key enzymes in monolignol biosynthesis, were upregulated in the infected plants (Table 2). Of the gene families encoding key enzymes in lignin biosynthesis—namely cinnamyl alcohol dehydrogenase (CAD), which catalyzes the interconversion of aldehyde and alcohol, and peroxidase, which catalyzes the polymerization of lignin—some genes were upregulated and some were downregulated after SPFMV and SPCSV infection. Genes encoding two enzymes involved in general phenylpropanoid metabolism, phenylalanine ammonia lyase (PAL), and cinnamate 4-hydroxylase (C4H) were significantly downregulated. However, genes encoding 4-coumarate-COA ligase (4CL), which catalyzes the biosynthesis of 4-coumaroyl-CoA, the common precursor of the monolignol and flavonoid biosynthetic pathways, were upregulated. Unigene0008344, which encodes aspartate aminotransferase (EC 2.6.1.1), an enzyme that catalyzes the interconversion of phenylalanine and phenylpyruvate, was upregulated. This result indicates that the metabolic flux to flavonoid and lignin biosynthesis was mainly reduced in the infected plants.

### 2.9. SPVD Alters the Expression of Genes Involved in Carotenoid, Zeatin, and Linoleic Acid Metabolism

Similar to genes in the flavonoid metabolic pathway, genes encoding key enzymes controlling carotenoid biosynthesis were downregulated in the SPFMV and SPCSV-infected plants (Figure 6C). Of the DEGs related to zeatin biosynthesis, the gene encoding cytochrome P450 monooxygenase CYP735A was significantly upregulated in the infected sweet potato. CYP735A hydroxylates isopentenyl ribotides to form trans-zeatin ribotides, and is thus a key enzyme in cytokinin biosynthesis [21]. In addition, there were four DEGs encoding adenosine phosphate isopentenyltransferases (IPTs), which catalyze the first step of cytokinin biosynthesis. One of these genes was upregulated and the other three were downregulated in the infected plants.

Among the DEGs involved in linoleic acid metabolism, the triacylglycerol lipase gene *SDP1* was significantly upregulated in the infected plants. SDP1 was reported to be involved in membrane lipid homeostasis in Arabidopsis leaves [22], but the meaning of its upregulation in the infected sweet potato plants needs to be further investigated.

### 2.10. DEGs that Participate in the Response to SPVD

To elucidate sweet potato’s defense response against SPVD and to discover potential SPVD resistance mechanisms, we explored DEGs related to plant–pathogen interaction and defense response. Homologs of heat shock protein 70 (HSP70) and heat shock protein 90 (HSP90) genes, which have been reported to play key roles in resistance to viruses in plants [23,24,25], were significantly induced in SPFMV and SPCSV-infected sweet potato. The RPM1-interacting protein 4 (RIN4) gene homolog was also upregulated, as were unigenes encoding WRKY1, WRKY6, WRKY22, and WRKY40-like WRKY transcriptional factors. The upregulated expression of these WRKY genes was verified in the infected plants using qRT-PCR (Supplementary Materials Figure S2). In addition, we detected significant induction of a previously unreported leucine-rich repeat receptor-like serine–threonine protein kinase gene, Unigene0001450, which showed 95% sequence identity with XM_031273346.1 in GenBank. Furthermore, a previously unreported TIR-NBS-LRR-type disease resistance (R) protein gene (Unigene0003226) was expressed in S14 but not H14.

Other pathogen-related genes were downregulated in S14 when compared to H14. The downregulated genes included disease resistance protein RPM1 and salicylic acid binding protein 2 (SABP2) gene homologs, and surprisingly, a pathogenesis-related protein 2 (PR2) gene homolog. The downregulated expression of this *PR2* homolog in the infected plants was also verified using qRT-PCR (Figure S2). A putative *CC*-NBS-LRR type *R* gene (Unigene0026690, which showed 95% sequence identity with XM_031258539.1 in GenBank) was also downregulated in S14. Furthermore, one of the genes shown to be downregulated in the infected plants encodes the transcription factor TGACG-BINDING FACTOR 1 (TGA1), which plays important roles in plant immunity [26].

## 3. Discussion

Our survey of the distribution and spread of SPVD in 10 sweet potato varieties revealed several features of SPVD spread in the field, meaning some SPVD control strategies can be tailored accordingly. First, SPVD spread from the plants surrounding the central originally infected plants to distant plants, which might reflect the flight range and dispersal of insect vectors. This result indicates that after infected plants are removed from a field, the surrounding plants should be monitored to prevent long-distance dispersal of SPVD. Second, the number of symptomatic plants increased rapidly during the early stages of plant growth but only slowly at later stages. This result might be attributed to the small size of the plants and the ease of virus transmission by vectors in the early stages of growth, along with the fact that it takes longer for visible symptoms to develop on older, bigger plants [12]. However, we also observed that the number of symptomatic leaves increased rapidly in older plants, especially in Yushu No.2 variety, indicating that although the number of symptomatic plants increased slowly, the virus spread rapidly within infected plants.

These results show that for some varieties, the severity of SPVD in the field should be evaluated based on both incidence rate and the number of symptomatic leaves per plant. In addition, removal of infected plants was an effective method to prevent SPVD spread in the field, especially during the later stages of growth. Third, SPVD symptoms were first detected in newly emerging apical leaves. This disease pattern is consistent with the results of a previous study in sweet potato [11,12], but the reason for it is unclear.

The prevalence of SPVD was mainly determined by the SPVD resistance of the varieties, but it was also influenced by other factors. Graft inoculation is an effective method for assessing SPVD resistance [27]. In this study, grafting experiments showed low disease indices for Yushu No.4 and Yushu 12 plants, indicating that they are SPVD-resistant or -tolerant varieties. This resistance could explain the 0 infection rate observed for these two varieties over the two years of field experiments and in the friction inoculation experiments.

The disease indices detected for Yushu No.6 (small), Yushu No.2 and Xushu 22 (moderate), and Ningzishu No.1 (high) were also consistent with their small, moderate, and high SPVD incident rates, respectively, both in the field and in friction experiments. These results indicate that the spread of SPVD can be controlled using resistant varieties. Interesting, we obtained a high disease index (63.57) for graft-inoculated Ning 4-6, which identified it as an SPVD-susceptible variety, but no infected Ning 4-6 plants were detected in the field and friction experiments. This result was consist with the view that cultivars considered highly resistant to SPVD under field conditions can still become diseased when graft-inoculated [28]. Our results indicate that the SPVD resistance or susceptibility of a variety determined by graft inoculation would not directly represent its SPVD resistance or susceptibility under natural conditions, possibly because beyond the resistance of varieties, SPVD incidence rates under natural conditions could also be affected by weather [29], geographic factors [30], and other unidentified factors.

In our experiments, friction inoculation resulted in only 50% or lower incidence rates, indicating that SPVD was not well-transmitted by this method. Nevertheless, the results show that stems may be more susceptible to SPFMV and SPCSV infection than other organs of sweet potato. The relative ease of transmission to stems might be because SPCSV is phloem-limited [11]. Direct rubbing or mechanical friction on stems would facilitate SPCSV transmission into the phloem. SPCSV could then enhance the multiplication of SPFMV and increase the titer of SPFMV in non-phloem tissues, causing severe SPVD symptoms [11]. This series of events would also explain why systemic infection by graft inoculation was effective for SPVD transmission.

SPFMV is not confined to the phloem; it can accumulate in leaf and stem tissues and is usually observed in leaves [11]. Our previous work showed that the infected Yushu No.6 plants had a high level of SPFMV and low level of SPCSV [31], meaning that SPFMV was the dominant SPVD virus in this variety. Then, for Yushu No.6, the dominant SPFMV could possibly be transmitted through friction inoculation of leaves and cause SPVD, which could explain the high infection rates obtained by this method for this variety (Figure 4A,B). Furthermore, one way plants can resist viruses is to restrict their movement from cell to cell or through the vascular system [11,32]. This mechanism might explain why the sweet potato varieties that were resistant to SPVD spread in the field experiments also showed low SPFMV and SPCSV infection rates in the friction experiments.

Sweet potato is a vegetatively propagated crop [14], and systemic pathogens such as viruses can persist and spread over successive crop cycles through vegetatively propagated materials [6]. To test for this type of transmission, we harvested the storage roots of asymptomatic plants from our friction inoculation experiments, generated seedlings from them, and planted the seedlings in 2015. The resulting plants exhibited more SPVD symptoms and higher rates of SPFMV and SPCSV infection than was observed in 2014, indicating that the virus could be transmitted through friction inoculation and remain latent in the storage roots.

During vegetative propagation of sweet potato, cuttings are taken from vines and then transplanted. If the knives used in this process contact vines with SPVD, it would lead to large-scale dispersal of SPVD after the cuttings are transplanted. Even when the infected plants exhibit no symptoms and have low virus titers of SPFMV and SPCSV, seed sweet potato and cuttings need to be carefully screened for SPVD, and strategies to block systemic movement of the virus toward the shoot tips need to be developed.

Each variety we tested exhibited SPVD symptoms specific to that variety. This may be due to a number of reasons. First, different ratios of the two viruses accumulated in the plants of different varieties [31]. Second, our previous study showed that there might be two types of SPFMV strains (EA and RC) and one type of SPCSV strain (WA) existing in these tested sweet potato leaves based on cluster analysis [31], the presence of different isolates of the two viruses, and synergistic interactions between them, contributing to variation in the severity and symptoms of SPVD [33]. Third, differences in variety characteristics and viral responses may also contribute to differences in the appearance of infected plants. Nonetheless, observing and recording the characteristic symptoms of each variety, especially the variety-specific symptoms, and surveying SPVD according to the characteristic symptoms of varieties cultivated in the field will be an effective method for monitoring and controlling SPVD in sweet potato production.

Understanding the molecular mechanisms of plant–virus interactions can provide new insights into virus resistance, and can lead to the development of new approaches for breeding of resistant varieties. We compared the gene expression profiles of non-infected and infected Wanshu No.8 plants grown in the field. These experiments revealed how SPVD affects sweet potato plants and how the plants respond to SPVD under natural conditions.

Viral infections can cause structural changes in chloroplasts, reduce photosynthesis, and increase respiration in infected leaves [34,35]. Our analysis of downregulated genes in the infected sweet potato indicated that SPFMV and SPCSV infection impaired photosynthesis by suppressing the expression of antenna proteins, thereby impairing the light-harvesting complex, disrupting photosystem I and II reaction centers, and inhibiting photosynthetic electron transport. These results are consistent with previous reports, which showed reduced expression of genes involved in the overall photosynthetic pathway in SPVD-affected plants revealed using cDNA microarrays [12]. However, contrary to the previous report, we detected no differential expression of the Rubisco small subunit 2B gene. We also observed downregulation of genes related to both photosystem I and II, whereas the previous report stated that only photosystem II was affected by SPVD [12]. These inconsistencies might be attributed to changes in the pattern of gene expression over the course of SPFMV and SPCSV infection.

Viral infections alter carbohydrate allocation in host plants [35]. Virus movement from cell to cell increases the permeability of plasmodesmata to virus particles and tends to decrease sugar transport from leaves to sink tissues, resulting in the accumulation of carbohydrates in the leaves and a subsequent decrease of photosynthesis [36]. The observed differences in SWEET transcript levels in the infected plants compared to non-infected plants indicate that sucrose phloem loading is modulated by SPFMV and SPCSV infection [35].

When compared with non-infected plants, a reduction of starch degradation and sucrose cleavage was detected in the infected plants, consistent with the previous report that soluble sugar and starch accumulate in leaves where the virus is actively replicating, and high sucrose levels in infected tissues could contribute to the increased carbon availability to sustain viral replication [35].

Furthermore, our results revealed the increased expression of trehalose metabolism-related genes and reduced expression of pectin and cellulose biosynthesis-related genes. A high level of trehalose 6 phosphate (Tre6P) was detected in wheat (*Triticum aestivum*) leaves infected with the Mal de Río Cuarto virus (MRCV) [35]. A similar mechanism might be present in sweet potato; trehalose metabolism and signaling were induced in SPFMV and SPCSV-infected plants, thereby regulating starch turnover and sucrose levels [35]. Pectin and cellulose are cell wall polysaccharides. The cell wall is a primary barrier that pathogens have to penetrate to initiate the infection process [37,38]. SPVD may confine pectin and cellulose biosynthesis, and thus constrain plant growth.

Plants can exhibit changes in several different metabolic pathways in response to viral infections [39,40]. When compared to uninoculated or mock-inoculated plants, the expression of phenylpropanoid metabolism-related genes was upregulated or downregulated in plants that had been inoculated with pathogens, and the metabolites were observed to be increased or reduced in the pathogen-infected host plants [39,41,42,43,44], emphasizing that the secondary metabolites produced through the phenylpropanoid pathway play important roles in the plant defensive response [45,46,47]. In our study, the transcriptomes of sweet potato plants also exhibited obvious changes in the expression of genes involved in the phenylpropanoid metabolic pathway following SPVD.

The expression of most genes belonging to families encoding key enzymes in flavonoid biosynthesis was reduced. Thus, we hypothesized that flavonoids might function as an early barrier against SPVD, and that genes involved in the flavonoid biosynthesis pathway are suppressed when the plant’s resources are directed towards SPVD replication and transmission. However, members of the F5H and CCR gene families involved in the monolignol biosynthetic pathway exhibited significant upregulated expression in the infected plants, which accounts for the increased caffeyl aldehyde, sinap aldehyde, and 5-OH coniferyl alcohol contents, and decreased caffeic acid, sinapinic acid, and ferulic acid contents in the infected plants, indicating that particular classes of compounds are induced in response to SPVD. Monitoring changes in the expression of phenylpropanoid metabolic pathway genes from the early to later stages of SPFMV and SPCSV infection might help reveal the roles of phenylpropanoids in SPVD resistance in sweet potato.

Chlorophyll pigments and carotenoid were less abundant in the leaves of Tungro-infected rice (*Oryza sativa* L.) plants than in those of healthy plants [48], and the total carotenoid content was reduced by 43%, 16%, and 37% in the orange-fleshed sweet potato variety Resisto following infection with SPCSV, SPFMV, and SPVD, respectively, when compared with healthy plants [49]. Our results showed that the genes involved in carotenoid biosynthesis were downregulated in the SPFMV and SPCSV-infected plants, indicating that the reduced carotenoid contents observed in the infected plants might be attributed to downregulation of gene expression involved in carotenoid biosynthesis. Since carotenoids are essential pigments of photosystems and important signals for plant growth and development [50], a reduction in carotenoid biosynthesis would affect the photosynthetic capacity and growth of the infected sweet potato plants.

The comparative transcriptome analysis elucidated putative genes participating in SPVD defense in sweet potato. *Nicotiana tabacum* and Arabidopsis SABP2 had strong esterase activity in converting methyl salicylate (MeSA) to salicylic acid (SA) and play important roles in systemic acquired resistance (SAR) signal development [51]. Furthermore, Arabidopsis *TGA1* regulates the biosynthesis of two important SAR signals, salicylic acid (SA) and pipecolic acid (Pip) [26]. In sweet potato, the SABP2 and TGA1 homologs might play similar roles in signal development, and SPVD might trigger signals that inhibit *SABP2* and *TGA1* expression, and thereby suppress SAR in the host plants. The roles of *RIN4* and *RPM1* in the plant immune response and hypersensitive response (HR) have been well established [52], but the involvement of these genes in SPVD defense needs to be investigated further.

PR2 is a well-established marker of SA-mediated defense, including basal defense, R-mediated defense, and SAR [53]. In previous studies, rice stripe virus (RSV) infection strongly induced *PR2* expression in Arabidopsis [54], while yellow dwarf virus (YDV) infection induced *PR2* expression in wheat [55]. The downregulation of *PR2* expression in the SPFMV and SPCSV-infected plants detected in this study might indicate that this homolog has different functions in virus-infected sweet potato. PR, R, and receptor-like kinases are three classes of proteins with established functions in plant defense responses. Some DEGs encoding leucine-rich repeat proteins or receptor-like kinases were detected in our transcriptome analysis, but only a few DEGs were related to pathogenesis. Additional genes involved in SPVD defense may emerge along with further excavation and annotation of the sweet potato genome.

WRKY1, 6, 22, and 40 had previously been demonstrated to be important regulators of pathogen defense and plant immunity [56,57,58,59]. Thus, the roles of the *WRKY* genes identified in our comparative transcriptomic analysis in viral defense in sweet potato merit further investigation.

Small RNA-mediated RNA silencing is an efficient mechanism to prevent viral infection in plants [60]. However, similar to observations revealed using cDNA microarrays [12], established RNA-silencing-related genes did not show differential expression between non-inoculated and SPVD graft-inoculated sweet potato. Further study is needed to decipher the RNA silencing components and mechanisms in sweet potato and their involvement in virus defense.

In this study, characteristics of the spread and transmission of SPVD were revealed, and suggestions on SPVD control strategies were proposed accordingly. The transcriptional alterations in SPFMV and SPCSV-infected sweet potato compared with non-infected plants were demonstrated using comparative transcriptomic analysis. We delineated how SPVD influences sweet potato and how sweet potato responds to SPFMV and SPCSV infection, and also identified genes involved in the response to SPVD. These findings provide insights into the control and prevention of SPVD, the breeding of resistant varieties, and the further elucidation of the pathology of SPVD and the virus defense mechanisms in sweet potato.

## 4. Materials and Methods

### 4.1. Plant Materials

Ten varieties were evaluated: Yushu No.2, Yushu No.4, Yushu No.6, Yushu No.12, Yushu No.33, Xushu No.22, Nanshu No.88, Ning 4-6, Ningzi No.1, and Wanshu No.8. Ning 4-6 and Nanshu 88 are orange-fleshed sweet potato varieties, Ningzishu No.1 is purple-fleshed, and the remaining 7 are white- or cream-fleshed.

### 4.2. Field Experiments

Field experiments were conducted at the experimental station of the Sweet Potato and Potato Research Institute, Southwest University, Chongqing, China, from May to December in 2014 and 2015. The experiments were carried out in randomized block design with two replications (Supplementary material Figure S1A). Each of the 10 varieties used was planted in a single plot with six mounds; the mound size was 5 m long and 20.8 cm wide, and the spacing between mounds was 80 cm. For each plot, 16 plants—which were identified as being co-infected with SPFMV and SPCSV using a nitrocellulose membrane enzyme-linked immunosorbent assay (NCM-ELISA), developed by the International Potato Centre (CIP), Peru—were planted in the center of the plot, and 128 plants that had been identified as being healthy using NCM-ELISA and qRT-PCR [31] were planted around the central SPFMV and SPCSV-infected plants (Figure S1B). Guard rows were set around the experimental plots. The entire experimental block was 16 m wide and 38.4 m long, and the passageway between plots was 0.5 m wide. The layout of the field experiment is shown in Figure S1. Sweet potato plants were monitored for virus symptom development, and the number of symptomatic plants, number of symptomatic leaves per plant, the symptoms, and the distribution of symptomatic plants in each plot were recorded at 9-day intervals throughout the growth stage, from the date on which the first symptomatic plants were detected. The typical symptomatic plants were further analyzed using NCM-ELISA and qRT-PCR [31] to confirm SPFMV and SPCSV infection.

### 4.3. Friction Inoculation Experiments

Friction experiments were conducted in the greenhouse from May to December in 2014. Each of the 10 varieties was planted in a single plot with two mounds; the mound size was 5 m long and 20.8 cm wide, and the spacing between mounds was 80 cm. For each plot, 48 plants that had been identified as being healthy using NCM-ELISA and qRT-PCR [31] were planted. The entire experimental block size was 11 m wide and 11.2 m long, with the passageway between plots being 0.5 m wide. Two mounds of guard rows were set around the experimental plots. Friction inoculation of SPVD was performed as described by Liu et al. [61]. The 48 plants in each plot were divided into four sets and each set was inoculated with SPVD through friction of the top leaf, stem, branch, or branch leaf, respectively. The friction inoculation was performed on two occasions at 14-day intervals in July 2014. The number of symptomatic plants was recorded at 9-day intervals from the date on which the first symptomatic plant was detected. The storage roots were collected and planted in 2015 to further survey the latent virus.

### 4.4. Graft Inoculation

Grafting experiments were conducted in the greenhouse from June to December 2016. The experiment was laid out in a randomized block design with two replications. Seven varieties were used. For each variety, 20 healthy plants were planted, of which 10 were used as a control, and another 10 plants were grafted with SPVD plants using the hole insertion method reported by Wang et al. [27]. SPVD symptoms were assessed 15 days after grafting (DAG) and 30 DAG, and the disease index was calculated according to Wang et al. [27].

### 4.5. Plant Materials used for Transcriptome Analysis

Three SPFMV and SPCSV-infected and three non-infected plants of Wanshu No.8, denoted here as S14 and H14, respectively, were collected from the field for transcriptional profiling. The SPFMV and SPCSV infection status of all the S14 and H14 plants had been diagnosed using both NCM-ELISA and qRT-PCR [31].

### 4.6. RNA Extraction

Total RNA samples were extracted from leaf samples and residual DNA was digested using an RNAprep Pure Plant Kit with DNase I (DP432, Tiangen Biotech, Beijing, China), according to the manufacturer’s instructions. The total RNA samples were examined by agarose gel electrophoresis, and the concentration and quality of RNA were assessed with a NanoDrop ND-2000 spectrophotometer (Thermo Scientific, Waltham, MA, USA). RNA quality was verified using a 2100 Bioanalyzer RNA Nanochip (Agilent Technologies, Santa Clara, CA) and all samples had an RNA Integrity Number (RIN) of >8.5. The RNA samples were equivalently pooled for transcriptional profiling and qRT-PCR analysis. A total of 20 μg of RNA from each sample was used for cDNA library preparation.

### 4.7. Illumina Sequencing, Data Filtering, and De Novo Assembly

Construction of cDNA libraries and Illumina paired-end (PE) sequencing using the Hiseq2000 platform was performed at Genedenovo Biotechnology Co., Ltd., Guangzhou, China, according to the manufacturer’s instructions (Illumina, San Diego, CA). To obtain clean reads, all of the raw reads were filtered using the following process. First, reads that failed the built-in failed chastity filter in the Illumina software according to the relation “failed chastity ≤ 1”, using a chastity threshold of 0.6, in the first 25 cycles were excluded. Second, reads with adaptor contamination were discarded. Third, low quality reads were masked with ambiguous sequence “N”. Finally, reads with more than 10% Q < 20 were removed. Filtered reads were de-novo-assembled using Trinity software (ver. trinityrnaseq_r2013_08_14) with the paired-end method.

### 4.8. Unigene Expression Analysis and Differentially Expressed Gene (DEG) Identification

To quantify the expression of unigenes, the filtered reads were mapped to unigenes using bowtie2-2.1.0, and the unigene expression levels were calculated and normalized according to the reads per kilobase of exon model per million mapped reads (RPKM) method. Based on the RPKM values observed, genes differentially expressed between S14 and H14 were screened by comparing the two libraries based on the combined criteria of *q*-value (false discovery rate (FDR)) of < 0.01 and absolute fold change of RPKM > 2. The differential expression of target genes was validated using the qRT-PCR method described previously [20].

### 4.9. Functional Annotation

BLASTx alignment (similarity > 30%, E < 1.0 × 10^−5^) was performed between unigenes and sequences derived from public databases, including the UniProt database (http://www. ebi.ac.uk/uniprot), Conserved Domain database (CDD) (http://www.ncbi.nlm.nih.gov/cdd), Pfam database (http://pfam.xfam.org/), National center for biotechnology information (NCBI) Non-redundant Protein (Nr) database (http://www.ncbi. nlm.nih.gov), and Eukaryotic Orthologous Groups (KOGs) database (http://www.ncbi.nlm.nih.gov/COG), and the best-aligned sequences were used to determine the sequence direction of unigenes.

### 4.10. GO Term and KEGG Pathway Enrichment

Gene ontology (GO) annotation analysis of the unigenes was performed using the high-scoring BLAST hits in the Swiss-Prot and TrEMBL protein databases (E value < 1.0 × 10^−5^) using Blast2GO (http://www.blast2go.com) [62]. The unigenes were further classified based on GO classification. To assign the detected unigenes to biological pathways, Kyoto Encyclopedia of Genes and Genomes (KEGG) pathway annotation was conducted using the online KEGG Automatic Annotation Server (KAAS, http://www.genome.jp/kegg/kaas/). The DEGs were analyzed for GO category enrichment and KEGG pathway enrichment using AgriGO [63] and KAAS, respectively, using Fisher’s exact test and FDR correction.

## Figures and Tables

**Table 1:** Disease indices of seven sweet potato varieties after graft inoculation of sweet potato virus disease (SPVD).

Variety	Disease Index Calculated at 15 DAG	Disease Index Calculated at 30 DAG
Yushu No.2	12.14 ± 1.01abAB	17.86 ± 1.01cB
Yushu No.4	5.71 ± 0.00abcABC	10.00 ± 4.04cBC
Yushu No.6	2.86 ± 0.00bcdABCD	7.86 ± 3.03cdBC
Yushu 12	2.86 ± 0.00bcdABCD	5.71 ± 0.00cdBCD
Xushu 22	12.14 ± 1.01abAB	26.43 ± 3.03bB
Ning 4-6	35.00 ± 1.01aA	63.57 ± 11.11bA
Ningzishu No.1	23.57 ± 1.01aAB	75.00 ± 13.13aA

Data show mean ± SD. Different uppercase letters in the same column indicate significant difference at *P* < 0.01 levels by Duncan’s test. DAG, days after grafting.

**Table 2:** Differentially expressed genes (DEGs) involved in the phenylpropanoid metabolic pathway.

Unigene	Enzyme	log2 Ratio	Up/Downregulation
Unigene0031486	phenylalanine ammonia lyase (PAL, EC 4.3.1.24)	−6.31	Down
Unigene0032806	cinnamate 4-hydroxylase (C4H, EC 1.14.13.11)	−2.14	Down
Unigene0033816	chalcone synthase (CHS,EC 2.3.1.74)	−16.48	Down
Unigene0017175	chalcone isomerase (CHI, EC 5.5.1.6)	−2.10	Down
Unigene0033967	flavonoid 3’-hydroxylase (F3′H, EC 1.14.13.21)	−13.36	Down
Unigene0028067	flavanone 3-hydroxyrase (F3H, EC 1.14.11.9)	−16.43	Down
Unigene0036071	dihydroflavonol 4-reductase (DFR, EC 1.1.1.219)	−15.59	Down
Unigene0035228	anthocyanidin synthase (ANS, EC 1.14.11.19)	−16.52	Down
Unigene0005028	flavonol synthase (FLS, EC 1.14.11.23)	−11.39	Down
Unigene0014446	hydroxycinnamoyl-CoA: shikimate O- hydroxycinnamoyltransferase (HCT, EC 2.3.1.133)	−1.89	Down
Unigene0033185	caffeoyl-CoA 3-O-methyltransferase 5 (CCoAOMT, EC 2.1.1.104)	−1.56	Down
Unigene0029746	serine carboxypeptidase (EC 2.3.1.91)	−1.01	Down
Unigene0036646	β-glucosidase 40 (EC 3.2.1.21)	−1.87	Down
Unigene0015200	4-coumarate-COA ligase (4CL, EC 6.2.1.12)	1.53	Up
Unigene0001094	Ferulate-5-hydroxylase (F5H, EC 1.14.13)	11.81	Up
Unigene0016523	cinnamoyl-CoA reductase (CCR, EC 1.2.1.44)	1.24	Up

## Supplementary Materials

The following are available online at https://www.mdpi.com/2223-7747/9/4/492/s1: Figure S1: Layout of field experiment. (**A**) Layout of sweet potato varieties in the field experiment. (**B**) Plant distribution in a single plot. White and black spots represent healthy and SPFMV and SPCSV-infected sweet potato plants, respectively. Figure S2: Validation of gene expression using qRT-PCR. Gene expression of *PR2* (A), *WRKY1* (B), *WRKY6* (C), *WRKY22* (D), and *WRKY40* (E) in transcriptome analysis revealed by RPKM. Error bars indicate the standard deviation of two independent replicates. Relative expression (ΔCt) of *PR2* (F), *WRKY1* (G), *WRKY6* (H), *WRKY22* (I), and *WRKY40* (J) in H14 and S14 detected using qRT-PCR. Error bars indicate the standard deviation of three independent replicates. Table S1: Primers used for qRT-PCR validation of the gene expression in H14 and S14.
