# Supplementary material for: The Spread and Transmission of Sweet Potato Virus Disease (SPVD) and Its Effect on the Gene Expression Profile in Sweet Potato
Source: Plants (Basel). 2020 Apr 10;9(4):492. doi: 10.3390/plants9040492 (PMC7238082; doi:10.3390/plants9040492)
Supplement: Supplementary file 1 [file plants-09-00492-s001.zip › Supplementary files/Table S1.docx]

**Table S1.** Primers used for qRT-PCR validation of the gene expression in H14 and S14.

| Gene | Primer name | Primer sequence |
| --- | --- | --- |
| *PR2*  (Unigene 0013990) | PR2-qPCR-F | 5’-CATTTTGGACTGTTTAGCCCTATTG-3’ |
|  | PR2-qPCR-R | 5’-CTATATGGGTATGTAGGGAAGT-3’ |
| *WRKY1*  (Unigene0020775) | WRKY1-qPCR-F | 5’-TGCAGGTTTGTTCCTGTCCTGGGAA-3’ |
|  | WRKY1-qPCR-R | 5’-AGATGTTGCAGAGACCTGCAGGGAA-3’ |
| *WRKY6*  (Unigene0020777) | WRKY6-qPCR-R | 5’-GTCTTTTCTTAATATCCCTCCG-3’ |
|  | WRKY6-qPCR-R | 5’-CTCTTCCTTTCTTCTTCCTTAG-3’ |
| *WRKY22*  (Unigene0013209) | WRKY22-qPCR-F | 5’-TTGTAATAGCTCTTCGAGTG-3’ |
|  | WRKY22-qPCR-R | 5’-TGAGGTTGAAATGTTGGTTG-3’ |
| *WRKY40*  (Unigene0029135) | WRKY40-qPCR-F | 5’-CCAATGATAGTGCTGTTTTG-3’ |
|  | WRKY40-qPCR-R | 5’-CATATGAAGGAGAGCATAACC-3’ |
