# Supplementary figures and images for: The Spread and Transmission of Sweet Potato Virus Disease (SPVD) and Its Effect on the Gene Expression Profile in Sweet Potato
Source: Plants (Basel). 2020 Apr 10;9(4):492. doi: 10.3390/plants9040492 (PMC7238082; doi:10.3390/plants9040492)

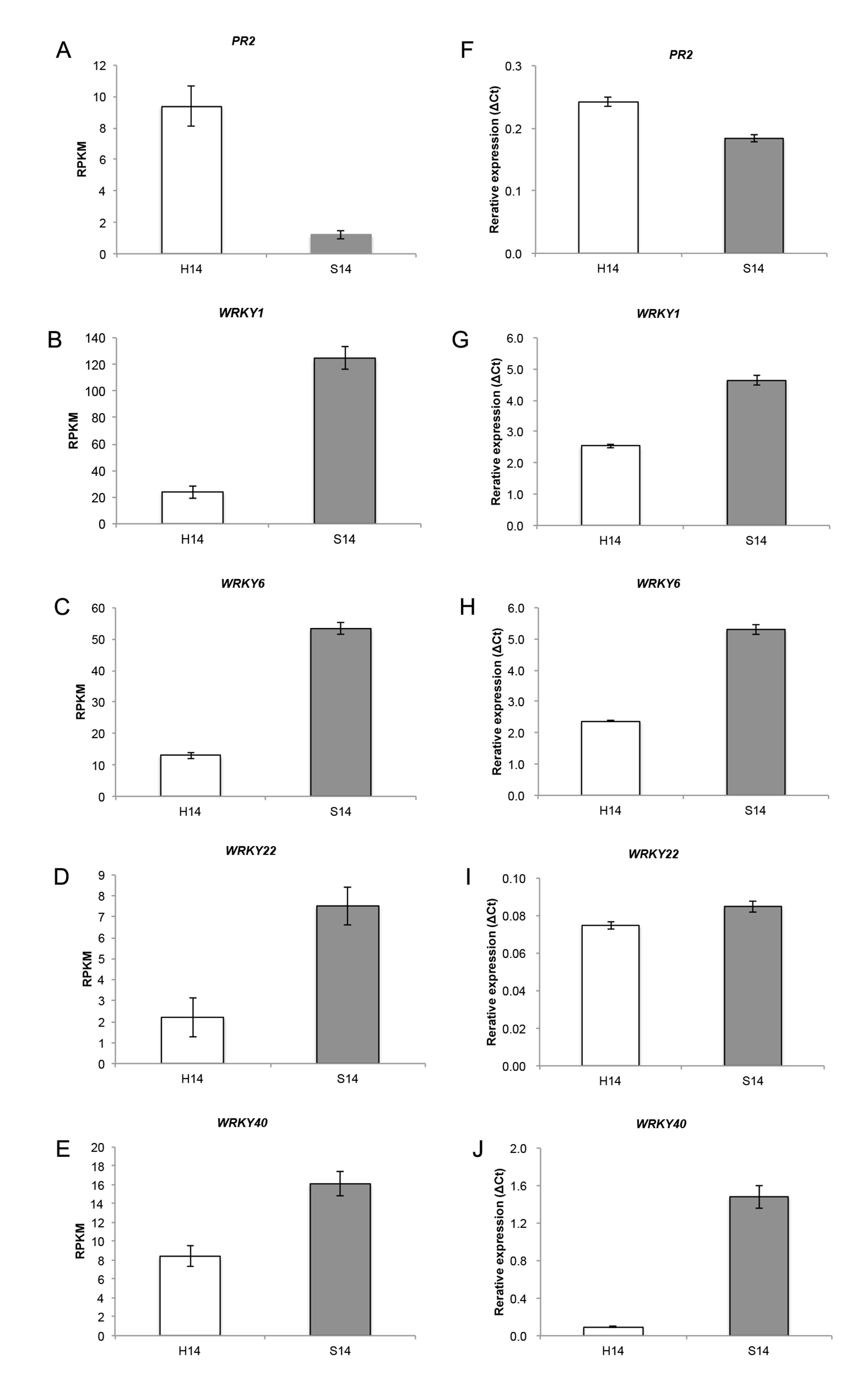

Supplement: Supplementary file 1 [file plants-09-00492-s001.zip › Supplementary files/Figure S2.tif]

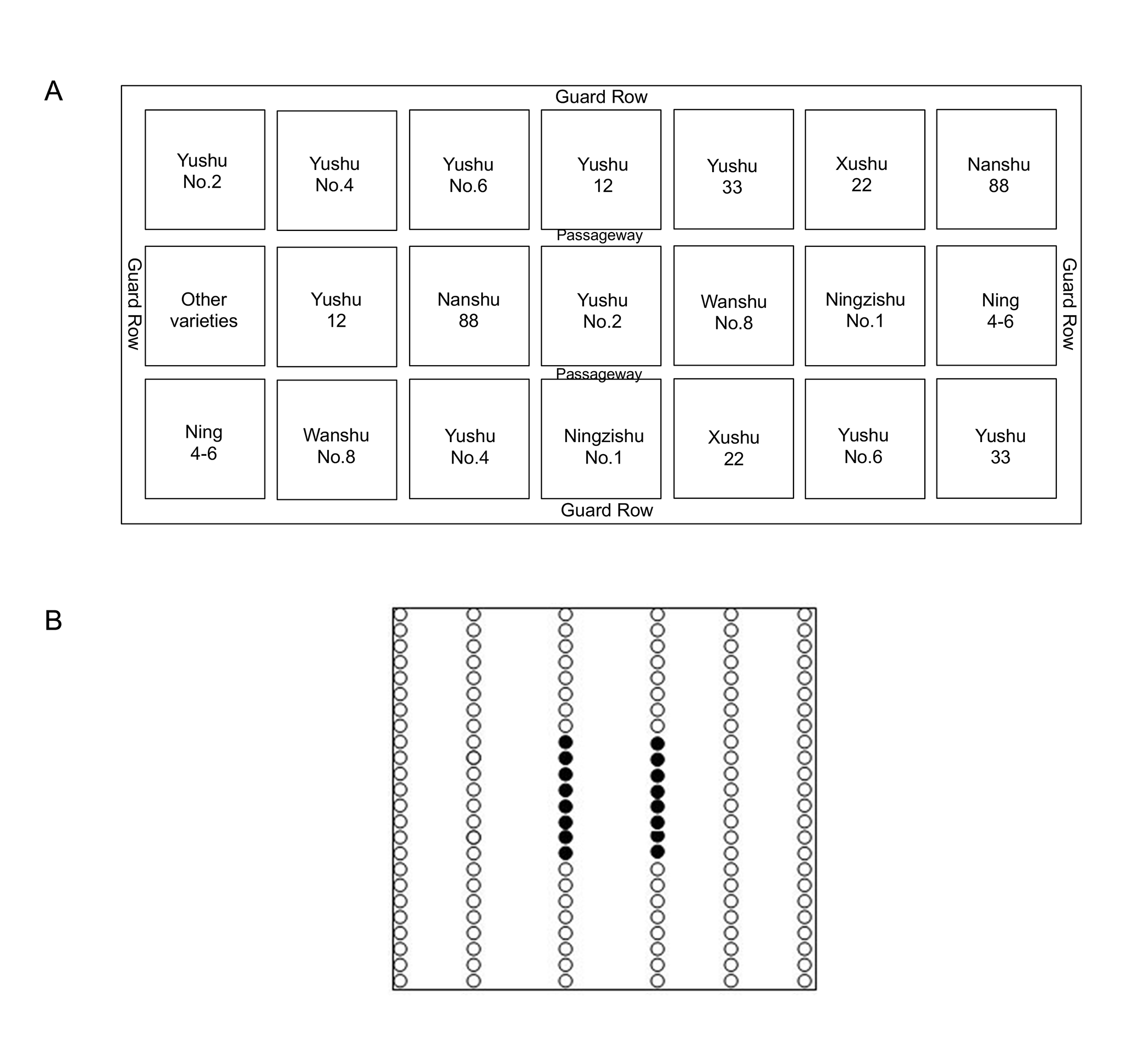

Supplement: Supplementary file 1 [file plants-09-00492-s001.zip › Supplementary files/Figure S1.tif]
